# Pathogenic Variations of Homologous Recombination Gene *HSF2BP* Identified in Sporadic Patients With Premature Ovarian Insufficiency

**DOI:** 10.3389/fcell.2021.768123

**Published:** 2022-01-31

**Authors:** Shan Li, Weiwei Xu, Bingying Xu, Shuchang Gao, Qian Zhang, Yingying Qin, Ting Guo

**Affiliations:** ^1^ Center for Reproductive Medicine, Cheeloo College of Medicine, Shandong University, Jinan, China; ^2^ National Research Center for Assisted Reproductive Technology and Reproductive Genetics, Shandong University, Jinan, China; ^3^ Key Laboratory of Reproductive Endocrinology of Ministry of Education, Shandong University, Jinan, China; ^4^ Shandong Provincial Clinical Medicine Research Center for Reproductive Health, Shandong University, Jinan, China; ^5^ Reproductive Hospital Affiliated to Shandong University, Jinan, China

**Keywords:** premature ovarian insufficiency, homologous recombination, HSF2BP, whole-exome sequencing, gene mutation

## Abstract

Premature ovarian insufficiency (POI) is defined as depletion of ovarian function before 40 years of age, which affects 3.7% of women in reproductive age. The etiology of POI is heterogeneous. Recently, with the widespread use of whole-exome sequencing (WES), the DNA repair genes, especially for those involved in meiosis progress, were enriched in the causative gene spectrum of POI. In this study, through the largest in-house WES database of 1,030 patients with sporadic POI, we identified two novel homozygous variations in *HSF2BP* (c.382T>C, p.C128R; c.557T>C, p.L186P). An *in vitro* functional study revealed that both variations impaired the nuclear location of HSF2BP and affected its DNA repair capacity. Our studies highlighted the essential role of meiotic homologous recombination genes in the pathogenesis of sporadic POI.

## Introduction

Premature ovarian insufficiency (POI) is defined by depletion of ovarian function before the age of 40 years, characterized by amenorrhea or oligomenorrhea and elevated serum level of follicle-stimulating hormone (FSH) (Webber et al., 2016). A recent meta-analysis found that 3.7% of women before 40 years of age are affected by POI (Golezar et al., 2019). The etiology of POI is heterogeneous. Genetic disorders, autoimmune disease, and iatrogenic factors account for nearly 50% of the patients, with the remaining half of the cases being idiopathic (Vujovic, 2009; De Vos et al., 2010). Nearly 20% of the cases have first- or second-degree relatives with POI. Therefore, genetic disorder is an indispensable factor in POI pathogenesis (Qin et al., 2015). Recently, with the widespread use of whole-exome sequencing (WES) in POI pedigrees, DNA repair genes, especially for those involved in the meiosis process, have been enriched in the spectrum of causative genes of POI (Jiao et al., 2018). However, the contribution of those genes in sporadic cases was rarely reported.

Meiosis is a crucial process during oogenesis. In the prophase of the first meiotic division, DNA double-strand breaks (DSBs) are generated by SPO11 (Keeney et al., 1997). After the modification by MRN, EXO1, and RPA (Soustelle et al., 2002; Lisby et al., 2004; Zakharyevich et al., 2010), the 3′-tailed single-strand DNA (ssDNA) of DSBs are recognized by BRCA2, RAD51, and DMC1, which execute the critical step of strand invasion, followed with homologous recombination (HR) and crossover formation (Barlow et al., 1997; Moens et al., 2002; Sharan et al., 2004). HSF2BP is a recently identified meiosis-specific protein, which interacted with BRCA2 and BRME1 to form a complex to mediate the recruitment of RAD51 and DMC1 to the meiotic recombination sites and facilitate the strand invasion (Zhang et al., 2020). *Hsf2bp* knockout female mice exhibited subfertility due to disruption of the recruitment of RAD51 and DMC1 onto ssDNA and delayed synapsis (Zhang et al., 2019). Recently, a homozygous missense variation in *HSF2BP* (HGNC:5226) was identified in a consanguineous family with POI (Felipe-Medina et al., 2020). However, the role of *HSF2BP* variations in sporadic POI pathogenesis was still unknown.

In the present study, through the variation screening of *HSF2BP* in the largest in-house WES database of 1,030 sporadic POI, two novel homozygous variations were identified. Functional studies showed both variants disturbed the nuclear localization of HSF2BP and affected its DNA repair capacity, highlighting the essential role of meiotic HR genes in the pathogenesis of sporadic POI.

## Materials and Methods

### Variation Screening of *HSF2BP* in the In-House Whole-Exome Sequencing Database of Premature Ovarian Insufficiency

A total of 1,030 females affected by idiopathic POI were recruited at the Center for Reproductive Medicine, Shandong University. The idiopathic POI was diagnosed by 1) primary or secondary amenorrhea or oligomenorrhea before 40 years of age; and 2) at least twice serum FSH >25 IU/L at an internal of 4–6 weeks. Patients with chromosomal abnormalities, ovarian surgery, chemo/radiotherapy, or autoimmune disease that might cause POI (such as systemic lupus erythematosus, Sjogren’s syndrome, rheumatoid arthritis, and autoimmune thyroiditis) were excluded.

Genomic DNA of the 1,030 patients was extracted from peripheral blood leukocytes using DNeasy Blood and Tissue Kit (Qiagen, Hilden, Germany). Exome sequences were captured with SureSelect Target Enrichment system for Illumina Paired-End Sequencing Library (Agilent Technologies, Santa Clara, CA, USA), and DNA sequencing was performed on Illumina HiSeq platform (Illumina HiSeq, San Diego, CA, USA). Reads were mapped to the hg19 reference genome, and variants were called and annotated using Genome Analysis Toolkit (GATK), ANNOVAR, and custom pipelines. The variants of *HSF2BP* were screened using the following strategies: 1) alter protein sequence (nonsense, missense, splicing-site variant, and coding indels); 2) allele frequencies <0.001 in the public databases 1000 Genomes, ESP6500, ExAC, and gnomAD; 3) variants predicted as deleterious variations by more than half of the software (SIFT, PolyPhen-2, MutationTaster, MutationAssessor, PROVEAN, and M-CAP); and 4) follow recessive inheritance pattern (homozygous or compound heterozygous). Then the variants were classified as pathogenic (P), likely pathogenic (LP), or variations of uncertain significance (VUS) according to the guidelines proposed by the American College of Medical Genetics and Genomics (ACMG) (Richards et al., 2015).

### Plasmids Construction and Mutagenesis

The wild-type HSF2BP-FLAG plasmids were generated by inserting human *HSF2BP* cDNA tagged with FLAG into pcDNA3.1 vector. One non-pathogenic single-nucleotide polymorphism (SNP) with relatively high allele frequency in gnomAD was chosen as a negative control. The mutant and polymorphic HSF2BP-FLAG plasmids were generated using QuikChange Lightning Site-Directed Mutagenesis Kit (Agilent Technologies) according to manufacturer’s instructions, with the primers listed in Supplementary Table S1 and confirmed by Sanger sequencing.

### Cell Culture

HeLa cells (human cervix carcinoma cell line) were cultured in Dulbecco’s modified Eagle’s medium/high glucose (Gibco, Grand Island, NY, USA) medium supplemented with 10% fetal bovine serum (Gibco) and 1% penicillin–streptomycin (Gibco). KGN cells (human granulosa cell line) were cultured in Dulbecco’s modified Eagle’s medium/F-12 (Gibco) medium supplemented with 10% fetal bovine serum and 1% penicillin–streptomycin. The cells were cultured at 37°C with 5% CO_2_.

### Immunofluorescence

HeLa cells and KGN cells were cultured in 24-well plates and transiently transfected with wild-type, mutant, and polymorphic HSF2BP-FLAG plasmids with the use of Lipofectamine 3000 (Invitrogen, Carlsbad, CA, United States). After being cultured for 48 h, the cells were divided into three groups: without treatment, treated with etoposide (ETO; 5 μg/ml, Solarbio, Beijing, China) for 1 h, and treated with mitomycin (MMC; 500 ng/ml, Sigma-Aldrich, St. Louis, MO, United States) for 8 h. Then, the treated cells were cultured with a normal medium and recovered for a specific time at 37°C. Then, the cells were fixed with 4% paraformaldehyde (Solarbio) for 20 min at room temperature. Then the slides were permeabilized with 0.3% Triton X-100, blocked with 10% bovine albumin for 1 h, incubated with anti-FLAG antibody (1:500 dilution, CST14793, Cell Signaling Technology, Danvers, MA, United States) at 4°C overnight, followed with incubation with fluorescent secondary antibody for 1 h, and then treated with antifade mounting medium with DAPI (Beyotime, Shanghai, China). Intervening phosphate-buffered saline (PBS) washes were performed after incubation when necessary. Images were captured with an Olympus (Tokyo, Japan) BX61 microscope. The nuclear vs. cytoplasmic ratio of the HSF2BP proteins in HeLa cells and KGN cells was quantified by ImageJ software.

### DNA Damage and Repair Assay

HeLa cells were cultured and transfected with wild-type or mutant HSF2BP-FLAG plasmids as mentioned above. At 48 h after transfection, the cells were incubated with ETO (5 μg/ml) for 1 h or MMC (500 ng/ml) for 8 h at 37°C to induce DNA damage. Then the culture medium with ETO or MMC was removed, and the cells were cultured with normal medium for a specific time at 37°C. The phosphorylation of the Ser-139 residue of histone variant H2AX (γH2AX; 1:1,000 dilution, Sigma-Aldrich, 05-636), which was a sensitive marker for DNA damage, was analyzed by Western blotting. Three independent experiments were conducted. Moreover, to compare the change of γH2AX level, we quantified the grayscale scores of Western blotting bands using ImageJ software and normalized the levels of γH2AX to the β-actin loading control in each group. The grayscale scores of γH2AX in the cells treated with ETO or MMC and recovered at specific time points were divided by those of untreated cells in each group. We compared the relative grayscale score between the wild-type and mutant groups at specific time points.

## Results

### Homozygous Missense Variations of *HSF2BP* Identified in Premature Ovarian Insufficiency Patients

**FIGURE 1:**
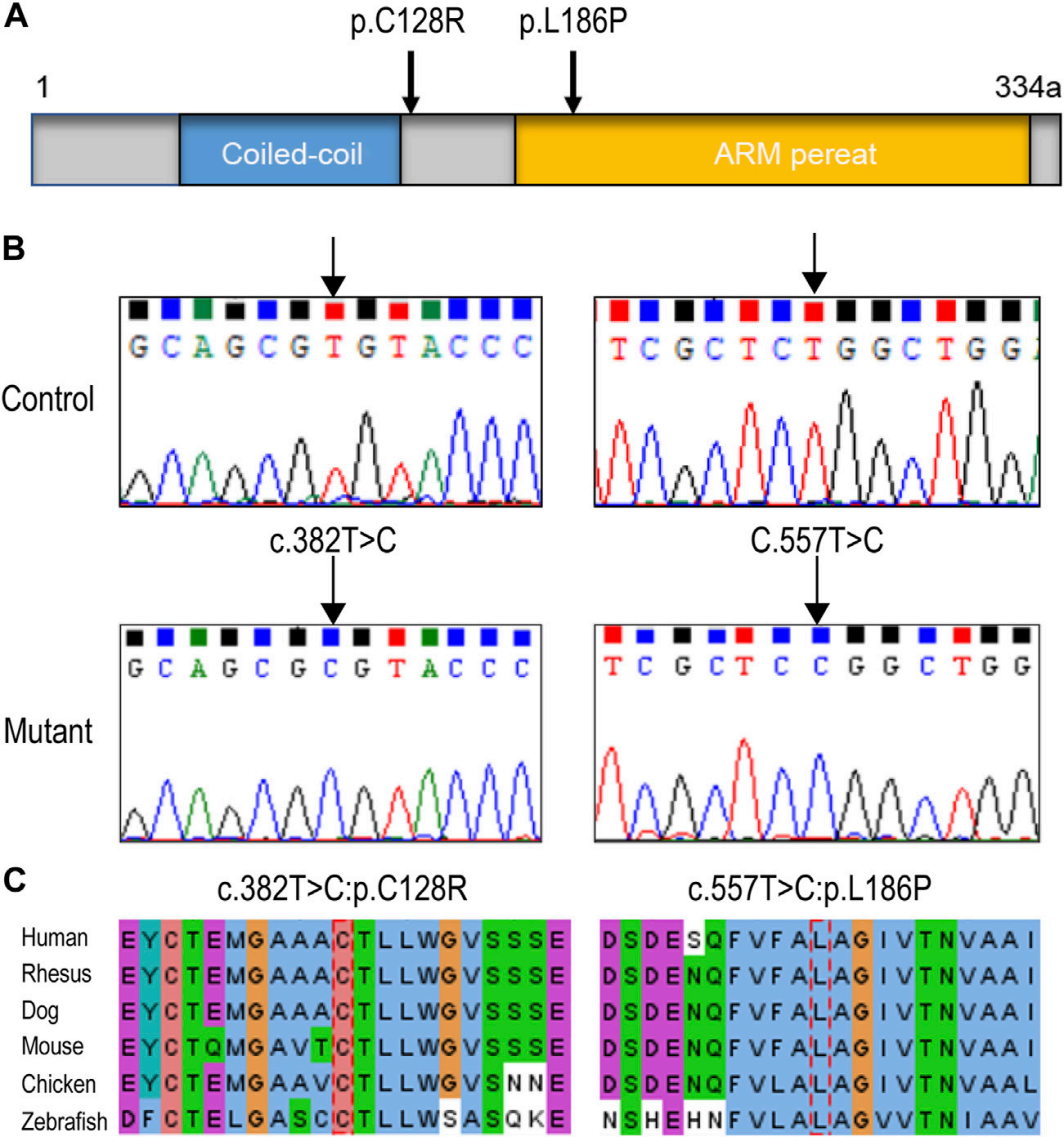
*HSF2BP* variations identified in premature ovarian insufficiency (POI) patients. **(A)** The structure of HSF2BP protein and the localization of the two variations. **(B)** The DNA sequence chromatograms of two variations identified in present study. **(C)** The amino acid sites of the two variations were highly conserved among species.

Through variation screening in the in-house WES database of sporadic POI, eight variations in *HSF2BP* (GenBank: NM_007031.2) have been identified. However, only two homozygous missense variations (following recessive inheritance pattern) were found, c.382T > C (p.C128R) and c.557T > C (p.L186P), which were with frequencies below 0.001 in the public databases 1000 Genomes, ExAC, and gnomAD, and were absent in the genomics database of 114,783 normal Han Chinese individuals (PGG.Han, http://www.pgghan.org). Both variants were confirmed by Sanger sequencing (Figure 1B), and the two amino acid sites were highly conserved among species (Figure 1C). According to the ACMG guideline, the two variants were classified as VUS.

### Clinical Characteristics of Variation Carriers

Patient POI-1, who carried *HSF2BP* p.C128R, experienced menarche at 11 years old, underwent irregular menstrual cycle (2–12 months per menstrual cycle) since 13 years old, and suffered amenorrhea at 24 years of age. Patient POI-2, who carried *HSF2BP* p.L186P, experienced menarche at 16 years old, followed by an irregular menstrual cycle that ceased at the age of 18. Ultrasound examination showed the two patients had atrophic ovaries without follicles (Table 1).

**TABLE 1:** Clinical characteristics of sporadic POI patients carrying *HSF2BP* variations.

Patient	*HSF2BP* variations				Menstrual history		FSH (IU/L)	LH (IU/L)	E_2_ (pg/ml)	Left ovary (cm × cm)	Right ovary (cm × cm)
	Variation type	Nucleotide variation	Amino acid variation	ACMG classification	Age at menarche (year)	Age at amenorrhea (year)					
POI-1	Homozygous	c.382T>C	p.C128R	LP	11	24	79.09	21.63	5.0	1.4 × 0.5 Follicle: 0	Invisible
POI-2	Homozygous	c.557T>C	p.L186P	LP	16	16	71.37	38.61	<5.0	Invisible	Invisible

Note. LP, likely pathogenic; FSH, follicle-stimulating hormone; LH, luteinizing hormone; E_2_, estradiol; POI, premature ovarian insufficiency; ACMG, American College of Medical Genetics and Genomics.

### Variations Impaired the Nuclear Localization and DNA Repair Capacity of *HSF2BP*

**FIGURE 2:**
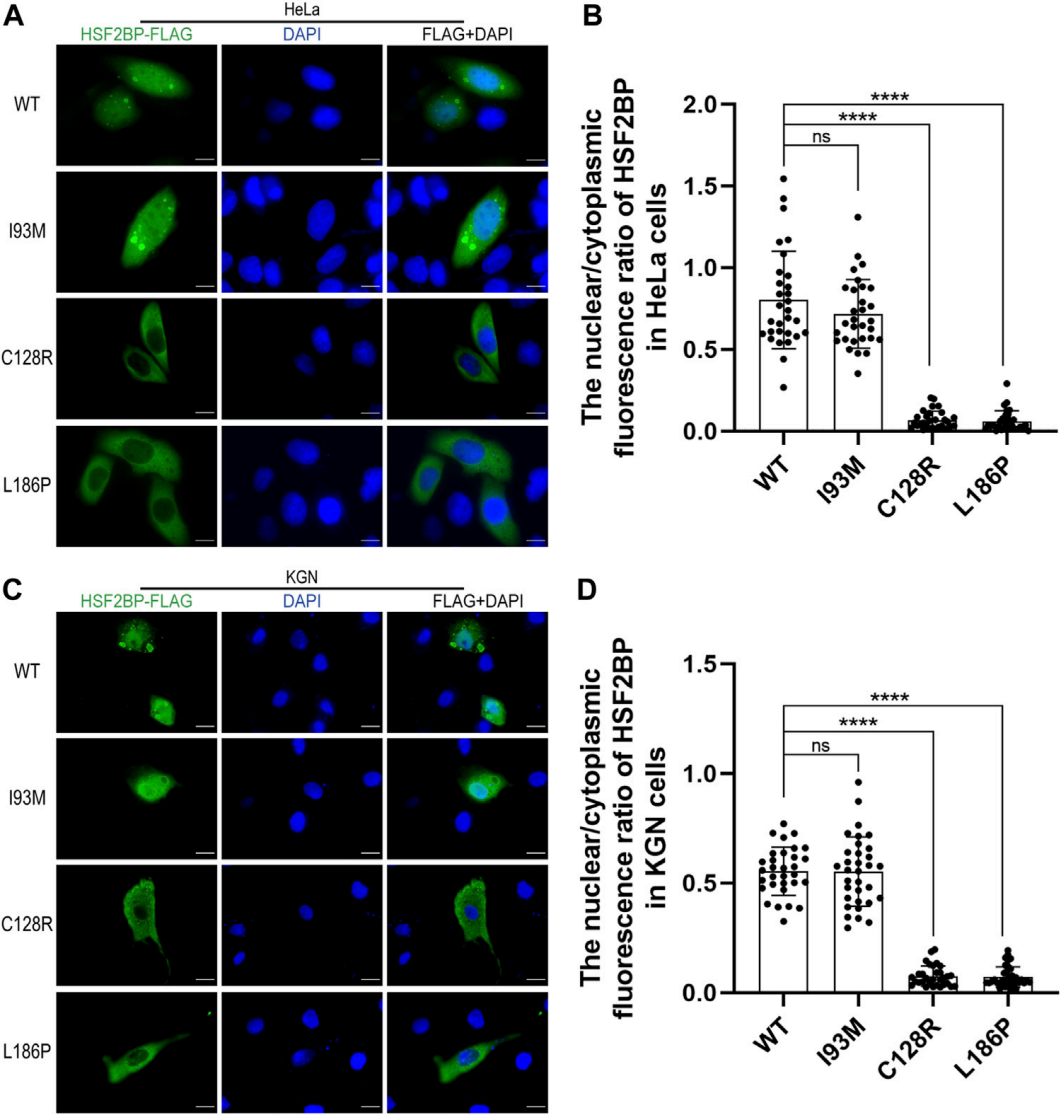
The two variations impaired the nuclear localization of HSF2BP. **(A,C)** Immunofluorescence against FLAG (green) in HeLa cells **(A)** and KGN cells **(C)** overexpressing wild-type (WT) or mutant HSF2BP (I93M, C128R, and L186P) plasmids. The mutant I93M was a polymorphism variant used as a control here. The nuclei were counterstained with DAPI (blue). Scale bars are 10 μm. **(B,D)** The nuclear vs. cytoplasmic ratio of HSF2BP proteins in HeLa cells **(B)** and KGN cells **(D)**. The differences of nuclear vs. cytoplasmic ratio between wild-type and mutant C128R or L186P groups were statistically significant. ****p < 0.0001.

**FIGURE 3:**
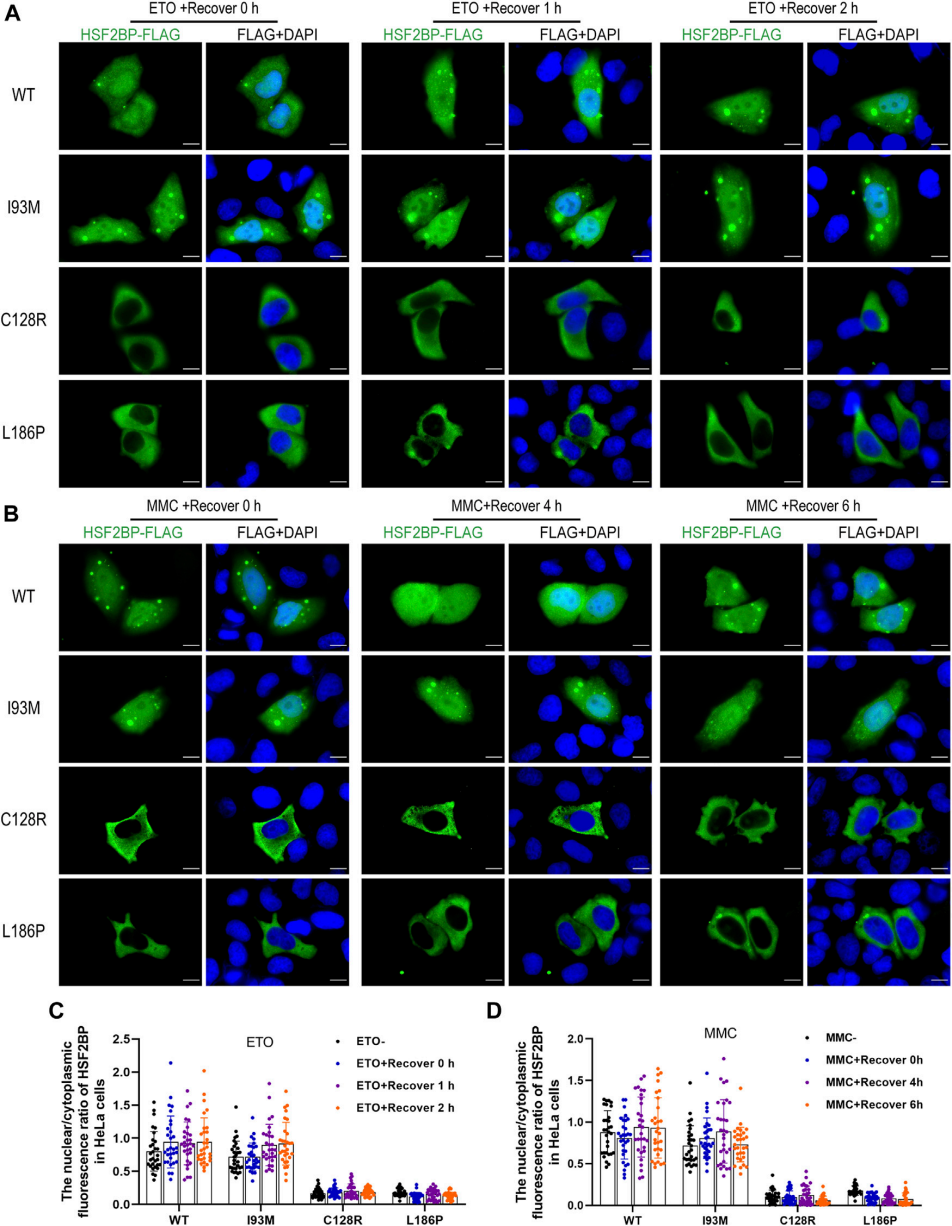
The disturbed nuclear localization of mutant HSF2BP was not affected by DNA damaging agents. **(A,B)** Immunofluorescence against FLAG (green) in HeLa cells overexpressing wild-type (WT) or mutant HSF2BP (I93M, C128R, and L186P) plasmids with etoposide (ETO) **(A)** or mitomycin (MMC) **(B)** treatment. The nuclei were counterstained with DAPI (blue). Scale bars are 10 μm. **(C,D)** The nuclear vs. cytoplasmic ratio of HSF2BP proteins in HeLa cells with ETO **(C)** or MMC **(D)** treatment and recovery at specific time points.

The wild-type or mutant HSF2BP-FLAG plasmids were transfected into HeLa cells and KGN cells, and the immunofluorescence against FLAG was performed to test the cellular localization of protein HSF2BP. The polymorphic variant I93M (rs200533753) was used as the negative control. The wild-type HSF2BP and I93M were expressed in both the cytoplasm and nucleus of HeLa and KGN cells. However, the signals of two mutant proteins (C128R and L186P) localizing in the nucleus were significantly lower than those of the wild-type protein (Figure 2). Moreover, after treatment with ETO or MMC, the localization of mutant proteins in cells was not changed (Figure 3). These results suggested that the variations C128R and L186P impaired the nuclear location of HSF2BP, where its DNA repair function was proposed to be performed, and the disturbed nuclear localization of mutant HSF2BP was not affected by DNA damaging agents.

**FIGURE 4:**
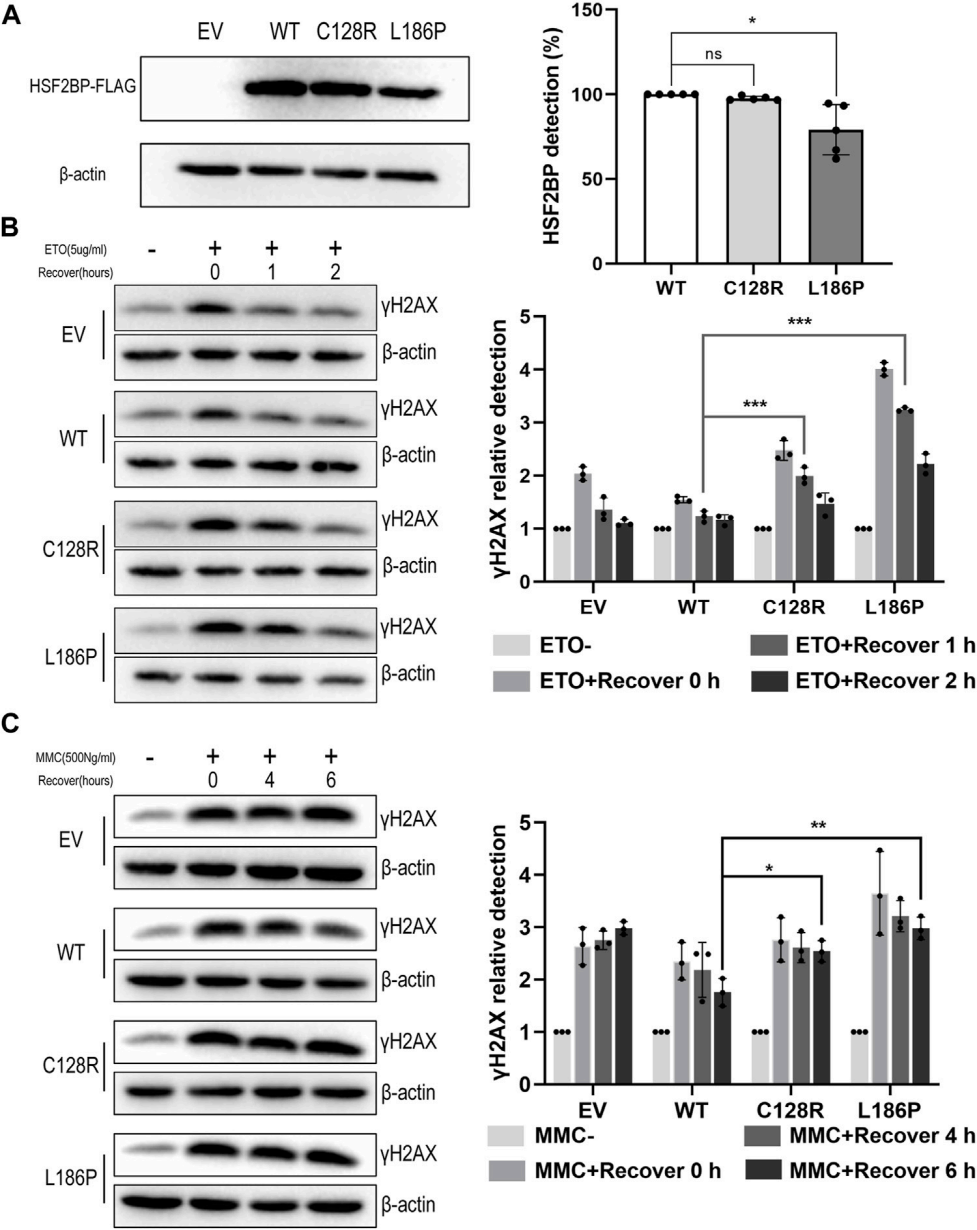
The variation C128R and L186P impaired the DNA repair capacity of HSF2BP. **(A)** The expression level of wild-type (WT) and mutant HSF2BP-FLAG (C128R and L186P) were tested by Western blotting. β-Actin was used as the loading control. Graph on the right represents the relative quantification of the immunoblotting. *p < 0.05. **(B,C)** The γH2AX level was detected by Western blotting in HeLa cells overexpressing empty vector (EV), wild-type (WT), or mutant HSF2BP-FLAG (C128R and L186P) with or without etoposide (ETO) **(B)** or mitomycin (MMC) **(C)** treatment. The relative grayscale scores of γH2AX in the cells overexpressing mutant HSF2BP (C128R and L186P) were compared with those overexpressing wild-type (WT) HSF2BP at specific recovery time points after ETO or MMC treatment (the grayscale of no treatment was considered as one in each group). β-Actin was used as the loading control. *p < 0.05, **p < 0.01, ***p < 0.001.

To elucidate whether the two variations impaired the DNA repair capacity of HSF2BP, DNA damage assays were performed. The result showed that the level of γH2AX in HeLa cells overexpressing wild-type HSF2BP increased immediately after ETO treatment and decreased to the untreated level after recovery for 1 h. Whereas, the γH2AX level in cells overexpressing mutant HSF2BP was significantly higher than that in the wild-type group after recovery for 1 h (Figure 4B). Similar results were observed in the MMC treatment groups, in which the γH2AX level in cells overexpressing mutant HSF2BP was significantly higher than that in wild-type cells after recovery for 6 h (Figure 4C), indicating that the mutant HSF2BP had impaired DNA repair capacity. Considering that HSF2BP was especially expressed in germ cells, the ultimately repaired DNA damage, demonstrated by decreased γH2AX level after 2 h of recovery in ETO-treated cells, might be caused by the endogenous DNA repair mechanism of HeLa cells. Then, with those functional studies, both variations were classified as LP according to the ACMG guideline (Table 1).

## Discussion

In the present study, two novel pathogenic homozygous variations of *HSF2BP* were identified in two patients with sporadic POI. Functional studies revealed the variations adversely affected the DNA repair capacity of HSF2BP due to disturbed nuclear localization, highlighting the essential role of meiotic HR genes in the pathogenesis of sporadic POI.

HSF2BP is an interactor of the heat shock response transcription factor HSF2 and is predominately expressed in germline cells (Yoshima et al., 1998; Brandsma et al., 2019). Recent studies found that HSF2BP interacted with BRME1 to form a complex that facilitated BRCA2-mediated recruitment of RAD51 and DMC1 onto ssDNA and strand invasion, which was essential for meiotic HR and crossover formation (Zhang et al., 2020). The knockout female mice of *Hsf2bp* exhibited a dramatically reduced number of oocytes (Zhang et al., 2019). In a consanguineous family with three cases of POI, homozygous variation p.S167L in *HSF2BP* was firstly reported. The point mutant mice *Hsf2bp* ^S167L/S167L^ showed reduced fertility with smaller litter sizes. With the observation that homozygous oocytes had a reduced number of RAD51/DMC1 foci on DSBs and subsequent reduction in the number of crossovers, *HSF2BP* p.S167L was assumed to cause POI by altered meiotic recombination (Felipe-Medina et al., 2020).

In the current study, with the in-house WES database of sporadic POI, two novel homozygous missense variations in *HSF2BP* (c.382T>C, p.C128R; c.557T>C, p.L186P) were identified, providing the evidence of *HSF2BP* variations participating in the pathogenesis of sporadic POI. A previously reported variation p.S167L resulted in the reduced protein level of HSF2BP and subsequent insufficient BRME1, RAD51, and DMC1 localizing at DSBs during meiotic recombination (Felipe-Medina et al., 2020). However, our *in vitro* studies merely found a modest reduction protein expression level of *HSF2BP* p.C128R and p.L186P (Figure 4A), indicating that their pathogenic effects on HSF2BP were different from those of p.S167L. Intriguingly, the hypothesis was confirmed by the observation that HSF2BP protein with variation p.C128R or p.L186P was barely transferred into the nuclei. A further study demonstrated that the DNA repair capacities of mutant HSF2BP were lower than those of the wild type. Therefore, *HSF2BP* p.C128R and p.L186P might lead to dysfunctional oogenesis by inefficient meiotic recombination due to disturbed nuclear localization of HSF2BP. Moreover, with the observation that the cells overexpressing mutant HSF2BP had increased sensitivity to DNA damaging agents, the role of accumulated DNA damage in somatic cells cannot be excluded in the pathogenesis of POI, especially for the cells during early embryo development (Yuen et al., 2012).

The establishment of the ovarian follicle pool is initiated by the primordial germ cells (PGC) migrating to the genital ridge; after PGC proliferation and meiosis prophase I, oocytes arrest at the diplotene stage in the form of primordial follicles, which determined the ovarian reserve (Baerwald et al., 2012). Meiotic HR is essential for genetic stability and diversity of oocytes, which also ensure the proper separation of homologous chromosomes during division (Baudat et al., 2013). Meiotic defects would cause accelerated oocytes apoptosis, resulting in diminished ovarian reserve even POI (Handel and Schimenti, 2010). Recent studies with next-generation sequencing extremely expanded the spectrum of POI candidate genes (França and Mendonca, 2020). Intriguingly, the genes involved in meiotic processes were intensively reported, such as DSB end processing genes *EXO1* (HGNC:3511), *MND1* (HGNC:24839), and *MEIOB* (HGNC:28569) (Caburet et al., 2019; Jolly et al., 2019; Luo et al., 2020); HR genes *RAD51* (HGNC:9817), *BRCA2* (HGNC:1101), *MSH4* (HGNC:7327), and *MSH5* (HGNC:7328) (Carlosama et al., 2017; Guo et al., 2017; Qin et al., 2019; Luo et al., 2020); and synaptonemal complex genes *SYCE1* (HGNC:28852), *C14ORF39* (HGNC:19849), and *STAG3* (HGNC:11356) (de Vries et al., 2014; Jaillard et al., 2020; Fan et al., 2021). The identification of *HSF2BP* further highlighted the essential role of meiotic HR genes in the pathogenesis of POI, for both familial and sporadic cases.

Intriguingly, recent studies found that the somatic cells overexpressing HSF2BP were sensitive to DNA interstrand crosslink, which increased cancer susceptibility (Brandsma et al., 2019; Sato et al., 2020). However, the two patients in the present study had no history of tumors at the time of the investigation. That might be caused by the observation that the two variations did not influence the expression level and tissue localization of HSF2BP. Even so, longtime follow-up of tumors for the *HSF2BP* variation carriers should be suggested.

In conclusion, two novel pathogenic homozygous variations of *HSF2BP* were identified in sporadic POI cases, further expanded the spectrum of POI causative genes, and highlighted the essential role of meiotic HR genes in sporadic POI pathogenesis.

## Data Availability

The datasets presented in this study can be found in online repositories. The names of the repository/repositories and accession number(s) can be found below: SCV001832610.1; SCV001832609.1 (ClinVar, https://www.ncbi.nlm.nih.gov/clinvar/).
