# Supplementary material for: Pathogenic Variations of Homologous Recombination Gene HSF2BP Identified in Sporadic Patients With Premature Ovarian Insufficiency
Source: Front Cell Dev Biol. 2022 Jan 31;9:768123. doi: 10.3389/fcell.2021.768123 (PMC8841426; doi:10.3389/fcell.2021.768123)
Supplement: Supplementary file 1 [file Table1.DOCX]

Table S1. Primers for *HSF2BP* mutagenesis.

| Mutation | Primer | Sequence |
| --- | --- | --- |
| c.382T>C | Forward | 5'-CACAAGAGGGTACGCGCTGCTGCTCCC-3' |
|  | Reverse | 5'-GGGAGCAGCAGCGCGTACCCTCTTGTG-3' |
| c.557T>C | Forward | 5'-TCGTGACAATTCCAGCCGGAGCGAAAACAAACTGA-3' |
|  | Reverse | 5'-TCAGTTTGTTTTCGCTCCGGCTGGAATTGTCACGA-3' |
| c.279A>G  (rs200533753) | Forward | 5'-CTCCTTCTTCTCTCTCATGTTGTCGGCCTGCAC-3' |
|  | Reverse | 5'-GTGCAGGCCGACAACATGAGAGAGAAGAAGGAG-3' |

Note: underline indicates the site of mutagenesis.
